# A longitudinal study of sleep in university freshmen: facilitating and impeding factors

**DOI:** 10.1093/sleep/zsaf156

**Published:** 2025-06-09

**Authors:** Chun Siong Soon, Xin Yu Chua, Ruth L F Leong, Ju Lynn Ong, Stijn A A Massar, Shuo Qin, Kyra H M Chong, Jukka-Pekka Onnela, Michael W L Chee

**Affiliations:** Centre for Sleep and Cognition, Yong Loo Lin School of Medicine, National University of Singapore (NUS), Singapore, Singapore; Centre for Sleep and Cognition, Yong Loo Lin School of Medicine, National University of Singapore (NUS), Singapore, Singapore; Centre for Sleep and Cognition, Yong Loo Lin School of Medicine, National University of Singapore (NUS), Singapore, Singapore; Centre for Sleep and Cognition, Yong Loo Lin School of Medicine, National University of Singapore (NUS), Singapore, Singapore; Centre for Sleep and Cognition, Yong Loo Lin School of Medicine, National University of Singapore (NUS), Singapore, Singapore; Centre for Sleep and Cognition, Yong Loo Lin School of Medicine, National University of Singapore (NUS), Singapore, Singapore; Centre for Sleep and Cognition, Yong Loo Lin School of Medicine, National University of Singapore (NUS), Singapore, Singapore; Department of Biostatistics, Harvard University, Boston, MA, United States; Centre for Sleep and Cognition, Yong Loo Lin School of Medicine, National University of Singapore (NUS), Singapore, Singapore

**Keywords:** sleep timing, freshmen, well-being, time use

## Abstract

**Study Objectives:**

Establishing healthy sleeping habits is a challenge for many college students. We determined how academic schedules influenced sleep patterns across the semester, and whether these are modulated by place of residence and class start times.

**Methods:**

A longitudinal cohort study evaluated 638 freshmen over their first 20-week semester, comprising instructional, reading, examination, and vacation weeks. Sleep duration and timing were measured daily with sleep trackers (Oura Ring 3). Time use was reported through a smartphone app.

**Results:**

Six hundred and thirty-eight participants (mean [standard deviation] age, 20.3 [1.3] years; females, 51.7 per cent) provided 64 642 nights of sleep data. Bedtimes were late (mean [standard deviation], 01:53 [71.76 min]). Weekday sleep duration increased after the midterm break (β = 2.74, *p* < .0001), arising from delayed wake times (β = 5.81, *p* < .0001). Pre-midterm, students woke up after class started on 42.6 per cent of days with early 08:00 classes, likely arriving late or skipping class. This percentage increased to 52.4 per cent post-midterm. Participants living on-campus (*n* = 357) had later bedtime and shorter weekday sleep than those off-campus (*n* = 281, bedtime: +37.26 min; *t*_629_ = 6.62; *p* < .0001; duration: −19.03 min; *t*_629_ = 6.36; *p* < .0001). They reported substantial in-person social and cocurricular activities on weekday nights (60 min in the 4 h before bedtime), and compositional data analysis indicated that spending more time on such activities would further delay their bedtime. Additional screen time showed the same effect for off-campus students.

**Conclusions:**

During university freshman year, when students had greater freedom to determine sleep timing, they slept more by waking up later, skipping early morning classes. However, other opportunities to lengthen sleep were not taken, highlighting the value of integrating contextual information with wearable data to understand real-world sleep behavior.

## Graphical Abstract

**Graphical Abstract:**
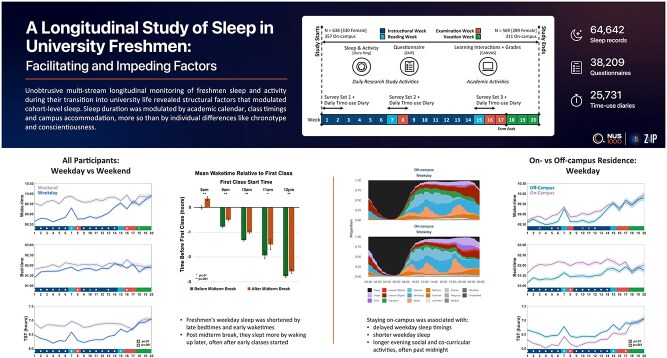

**Statement of Significance**

How does the structure of the academic term influence sleep patterns in college freshmen? Unobtrusive longitudinal monitoring revealed that despite competing time pressures, an expected early decline in weekday sleep duration was reversed in the latter half of the semester by students electing to wake up later and skipping early morning classes. In contrast, other factors expected to facilitate sleep, such as living on-campus, did not result in increased sleep duration. How freshmen exercise autonomy over their sleep is surprising; positive changes such as extending sleep can emerge from unexpected avenues (e.g. delaying weekday wake times), while more obvious opportunities (e.g. sleeping earlier) are not taken.

## Introduction

Starting college is a major milestone in a young adult’s life. The disruptive nature of this transition [1–5] creates opportunities to establish habits that could have lasting health consequences. Adequate sleep supports better mood, mental well-being, and learning [6–8], while poor sleep is a risk factor for multiple chronic health conditions [9,10], including anxiety and depression [11] that have increasingly high prevalence in college populations [12]. In many countries, sleep in college students is of lower quality or shorter duration than recommended [13–15]. Unique to this group is the combination of newfound autonomy to balance sleep with academic and social demands, more flexible schedules compared to high school students or working adults, and the ability to access pre-recorded study material, allowing them to learn at their own pace.

The advent of reliable [16,17], continuous long-term monitoring of sleep patterns [18] through widely available consumer health trackers has made it possible to collect large-scale sleep data cost-effectively. When combined with information on academic load [19,20], screen time [21], and mood [22], such tracking, demonstrated in smaller studies, has provided valuable time-course insights, surpassing the capabilities of conventional, episodic surveys [23–25]. This approach, adopted in the present study, offers a detailed and dynamic understanding of sleep patterns and their associated factors.

For example, tracking 49 freshmen across their first year of college, Bustamante and colleagues found that sleep duration fluctuated with academic demand, where students slept longer during vacation periods compared to instructional and examination periods [19]. On a day-to-day level, sleep is also affected by each day’s schedule. When college activities end late, sleep onset is delayed, and students obtain shorter sleep that night [26]. Over time, irregular school event schedules are associated with irregular sleep schedules [27]. Other sociodemographic or individual factors may also influence sleep and well-being, e.g. female undergraduates sleep earlier and longer than males [28], and on-campus residence is associated with later bedtimes and rise times [29]. With larger samples, using multiple streams of sleep and well-being data tracked over longer periods, structural and individual barriers and facilitators of sleep during transitional periods may be identified to help universities refine strategies to support healthy student life.

**Figure 1.**
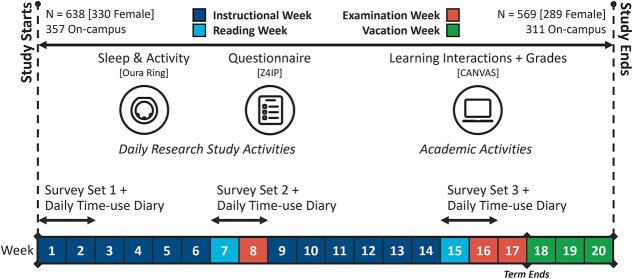
Schematic of study protocol. A multi-factor protocol was designed to maximize collection of objective and subjective data related to sleep, time use, emotional fluctuation, and academic performance of freshmen through their first semester while minimizing inconvenience to participants.

We tracked 638 freshmen across their first 20 weeks of college using validated sleep trackers, daily smartphone reports of sleep and well-being, and periodic time-use assessments (Figure 1). These data were integrated with information on students’ residence location to understand the barriers and facilitators of sleep during this critical period. We expected starting college to erode sleep time, but that time saved from not having to commute while staying on campus would be associated with longer sleep duration.

## Materials and Methods

### Participants

Freshmen enrolled into the National University of Singapore (NUS) in the 2023–2024 academic year were recruited prior to their first semester using flyers, posters, emails, and orientation camp announcements. Eligibility was assessed via an online demographic survey before obtaining consent. The NUS Institutional Review Board approved the protocols that were compliant with the Declaration of Helsinki. This report follows Strengthening the Reporting of Observational Studies in Epidemiology (STROBE) reporting guidelines for cohort studies. Participants were compensated up to SGD 355 (~USD 263), depending on the number of study components completed each day (see below).

### Protocol

#### Oura Ring

Participants were required to wear an Oura Ring 3 (Oura Health Oy, Oulu, Finland) for at least 20 h daily to track sleep. For each day, the longest sleep period between 18:00 the previous day and 17:59 the current day was considered the main sleep episode, and other sleep periods were considered naps. Further filtering based on Oura Ring wear time ensured accurate characterization of the broad range of sleep patterns under free-living conditions, particularly, nights with irregular, short sleep or absent sleep (Supplementary Methods and Supplementary Table S1). No imputation was performed for missing data. Days with overseas travel were excluded from all analyses.

#### Daily EMA: questionnaires and audio diary

Participants completed ecological momentary assessments (EMAs) daily between 20:00 and 23:59 h on our proprietary Z4IP app. Each assessment consisted of a questionnaire with questions repeated daily or weekly (Supplementary Methods).

#### Time-use diaries

In addition to daily measures, surveys and time-use diaries were completed during three fortnights at the beginning, middle, and end of the semester (Figure 1). Participants completed time-use diaries (Z4IP app) by selecting from a list of 20 activities (e.g. Sleep, Classes, Transport, Meals, and Others) for each 15-min window. In addition, each activity could be tagged as a “Group” activity, e.g. having a meal with other people. Each day’s activities were editable for up to 3 days. Only days with at least 75 per cent completion and three or more activity types were included for analyses [22,30].

### Data analysis

Weekly averages of weekday and weekend sleep parameters were obtained for each participant (minimum of one weekday and one weekend for a given week) from curated Oura Ring data. A linear mixed-effects model was specified to examine how weekday sleep timing and duration were affected by the fixed effects of instructional period (before vs. after the midterm break), week within each period, and their interaction, accounting for random intercepts for individual participants. A total of 500 participants with at least 4 weeks of data in each instructional period were included in this analysis.

To examine the association between class schedule and wake time, day-by-day wake times were stratified based on the time of the participants’ first class of the day. For each participant, the first class timing for each day of the week was determined by their official timetable (individual class schedules were largely similar across weeks). For each stratum, percentages of days with wake time occurring before and after each first class start time were computed.

For weekday vs. weekend comparisons within the same participant group, paired Student’s *t*-tests (two-tailed) were performed. Independent (two-tailed) *t*-tests were used to assess between-group comparisons for on-campus vs. off-campus residence.

Time-use behaviors in the 4-h period preceding individual weekday bedtimes were modeled using a compositional data analysis (CoDA) framework (R package “epicoda” [31]). This approach accounted for the codependent nature of time-use data by analyzing relative, rather than absolute, time spent in each activity [31,32]. Taking into consideration that some activities had a low probability of occurring close to bedtime, the reported activities were grouped as follows:

(1) Social (activities labeled as social, cocurricular, or tagged as “group”);
(2) Digital leisure;
(3) Self-study; and
(4) Other (any other activities).

Only nights with at least 75 per cent filled entries in the 4-h period were included. Missing time periods were assigned to other. Changes in bedtime associated with hypothetical reallocations of time (adding 1 h of social activities while proportionally reducing time spent on other activities), were modeled separately for the on-campus and off-campus groups. All effects were interpreted relative to the mean composition of the sample, representing the expected bedtime change for an average individual when reallocating time between activities (see Supplementary Methods for details).

## Results

### Sample characteristics

**Figure 2.**
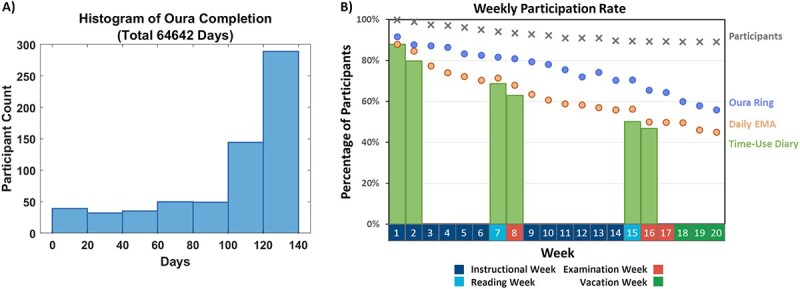
Participation rates for different data streams. (A) Histogram of Oura ring completion rate across participants. A total of 433 (67.9%) of participants provided 100 or more days of Oura data. (B) Weekly participation rate for different data streams (minimum of 1 weekday and 1 weekend per week). Despite a gradual decrease in participation rate as the semester progressed, all instructional weeks had Oura data from more than 450 participants.

The mean (standard deviation [*SD*]) age of the 638 participants was 20.3 (1.3) years; 330 were female (51.7 per cent); and 357 (56.0 per cent) lived on-campus (Table 1). A total of 69 participants withdrew over the course of the study. Study participants provided a total of 64 642 days (72.4 per cent) of Oura sleep recordings, and 38 209 days (42.8 per cent) of Z4IP EMA. In total, 17 726 days (66.2 per cent) of time-use diaries were recorded during the three fortnights required by protocol, and 8005 days outside these periods. Weekly participation rates are shown in Figure 2.

**Table 1.** Sample demographics and sleep characteristics grouped by residence

	Overall	On campus	Off campus
Characteristics	*n* = 638	*n* = 357 (56.0)	*n* = 281 (44.0)
Age, mean (*SD*), y	20.4 (1.3)	20.1 (1.2)	20.7 (1.4)
Sex			
Female	330 (51.7)	200 (56.0)	130 (46.3)
Male	308 (48.3)	157 (44.0)	151 (53.7)
Ethnicity			
Chinese	546 (85.6)	302 (84.6)	244 (86.8)
Malay	19 (3.0)	6 (1.7)	13 (4.6)
Indian	46 (7.2)	29 (8.1)	17 (6.1)
Others	27 (4.2)	20 (5.6)	7 (2.5)
Chronotype (MEQ)			
Score	44.7 (8.6)	44.9 (8.6)	44.6 (8.6)
Morning	14 (3.9)	9 (4.9)	5 (2.8)
Intermediate	217 (59.6)	108 (58.7)	109 (60.6)
Evening	133 (36.5)	67 (36.4)	66 (36.7)
Sleep, mean (*SD*)			
Days of data	101.3 (38.6)	96.9 (39.0)	106.9 (37.5)
Bedtime	01:53 (01:12)	02:04 (01:08)	01:38 (01:14)
Wake time	09:11 (01:09)	09:15 (01:08)	09:05 (01:09)
TST, h	6.5 (0.6)	6.4 (0.6)	6.6 (0.6)
Nap, h	0.3 (0.2)	0.3 (0.2)	0.2 (0.2)
Time use			
Days	40.4 (31.2)	36.3 (28.4)	45.6 (33.7)
EMA			
Days	59.9 (42.1)	54.2 (39.7)	67.1 (43.9)

MEQ, Morningness–Eveningness Questionnaire.

### Sleep patterns across the semester

**Figure 3.**
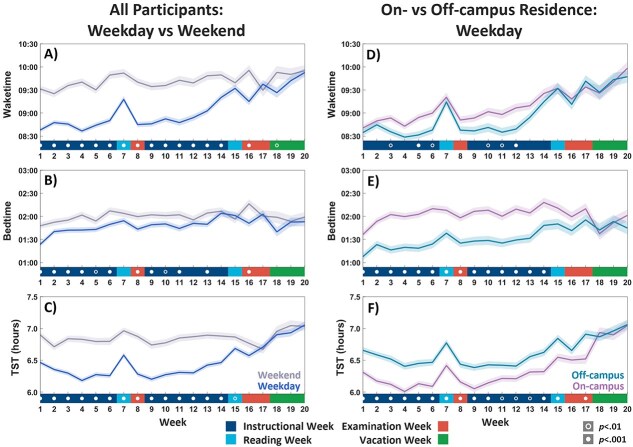
Weekly sleep trends of freshmen across their first semester. (A–C) Freshmen woke up earlier on weekdays but kept to a consistent late bedtime, resulting in weekday sleep curtailment. (D–F) Despite waking up later, those living on campus obtained less sleep due to relatively later bedtimes compared to those living off campus. Note the equalization of on- and off-campus groups during vacation as students return home in the latter case. Error bands represent standard error of the mean (*SEM*). Open (*p* < .01) and closed (*p* < .001) circles indicate significant differences (*t*-tests, uncorrected) for each week.

Weekly averages for weekday wake times ranged from 08:37 to 09:53 across the semester, while weekend wake times were relatively stable, ranging from 09:25 to 09:55 (Figure 3 and Supplementary Table S2). Bedtimes were more consistent than wake times across the semester, with weekly averages ranging from 01:24 to 02:04 on weekdays, and 01:47 to 02:16 on weekends.

A linear mixed-effects model revealed a significant interaction between instructional period and week (β = 5.56, 95% CI = 4.50% to 6.62%, *p* < .0001), indicating that the change in total sleep time (TST) across weeks differed before and after the midterm break (Figure 3). TST decreased over time before the midterm break (β = −2.82, 95% CI = −3.58% to −2.07%, *p* < .0001), but increased after (β = 2.74, 95% CI = 1.99% to 2.49%, *p* < .0001). A similar interaction was found for wake time (β = 5.81, 95% CI = 4.37% to 7.26%, *p* < .0001), but not for bedtime (β = −0.95, 95% CI = −2.35% to 0.45%, *p* = .183). Importantly, this upward trend was present independent of participant completion rates (see Supplementary Figure S1). These fluctuations in weekday sleep duration were not present for weekend sleep (Figure 3).

Inspection of trends in sleep efficiency and sleep onset latency showed minimal differences across the semester, while fluctuations in wake after sleep onset largely reflected differences in total time in bed (Supplementary Figure S2).

### Association between class start time and weekday wake time

**Figure 4.**
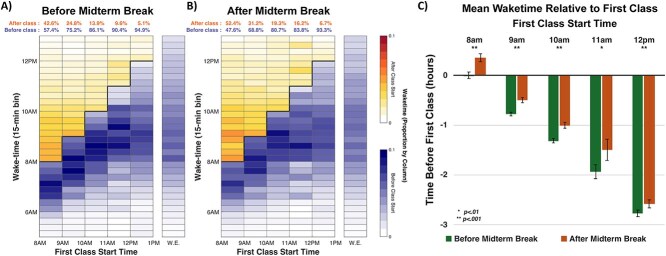
Wake time in relation to first class of the day before and after midterm break. (A, B) Heatmaps show the proportion of days when freshmen woke up before or after their first class of the day. The respective percentage of days with wake times before and after class start is shown above the heatmaps. Wake time distributions when classes started after noon resembled those on weekends (W.E.). After the midterm break, freshmen woke up later, likely skipping early classes more than before. (C) Bar chart shows mean (*SEM*) wake time before the first class start time. Asterisks indicate significant differences before vs. after midterm break (paired *t*-tests, uncorrected).

Wake times were closer to class start time for earlier classes and were further delayed after the midterm break (Figure 4). In particular, median wake time for 08:00 classes was 07:31 before the midterm break, but 07:59 after, just 1 min before class started, with 52.4 per cent of wake times occurring *after* class started [33]. In contrast, for 10:00 first class the median wake times were 08:33 before and 08:49 after the midterm break, i.e. most students were awake prior to class (before midterm, 87.4 per cent; after midterm, 81.4 per cent). When classes started at noon or later, wake times were similar to those observed on weekends. Similar trends were observed when the analysis was repeated using time-use diaries to determine first class of the day (Supplementary Figure S3).

### Association between campus residence and sleep

**Figure 5.**
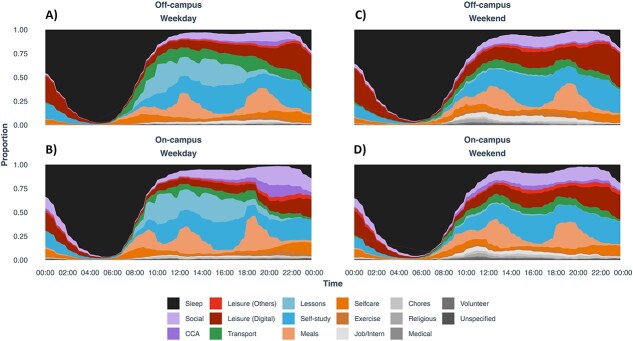
How freshmen living on- and off-campus spent their day during instructional weeks. (A) On weekdays, social and cocurricular activities (CCA) lasted late into the night for on-campus residents. (B) In contrast, off-campus students spent a substantial amount of time on digital leisure activities late in the night. (C, D) These weekday differences were muted on weekends.

On-campus residence minimized commuting, thereby potentially increasing TST (Figures 3D–F and 5 and Supplementary Figure S4). However, while on-campus residents woke up later on weekdays by 16.37 min (95% CI = 6.04% to 26.70%; *t*_629_ = 3.11; *p* < .005), bedtimes were delayed by 37.26 min (95% CI = 26.20% to 48.31%; *t*_629_ = 6.62; *p* < .0001), resulting in weekday TST being 19.03 min shorter than for those living off-campus (95% CI = −24.91% to −13.15%; *t*_629_ = −6.36; *p* < .0001). This finding remained even after naps were taken into consideration (−15.69 min; 95% CI = −21.55% to −9.83%; *t*_629_ = −5.26; *p* < .0001). Despite the greater opportunity for on-campus residents to nap in their dormitories, they did so for just 3.34 min more than off-campus students (95% CI = 1.12% to 5.57%; *t*_629_ = 2.95; *p* < .005) on weekdays, and 5.37 min more on weekends (95% CI = 2.59% to 8.15%; *t*_629_ = 3.80; *p* < .0005; Supplementary Figure S4).

Although the weekday–weekend difference in TST for those living on-campus (mean [*SD*], −34.87 min [44.27 min]) was greater (10.44 min; 95% CI = 3.53% to 17.36%; *t*_618_ = 2.97; *p* < .005) than those living off-campus (mean [*SD*], −24.54 min [40.85 min]), there was no difference in weekend sleep, even when naps (*t*_619_ = −1.10; *p* = .28) or vacation periods (*t*_381_ < 1) were factored in. Thus, group differences in weekday sleep duration and timing are unlikely to represent different sleep needs (Figure 3F).

**Figure 6.**
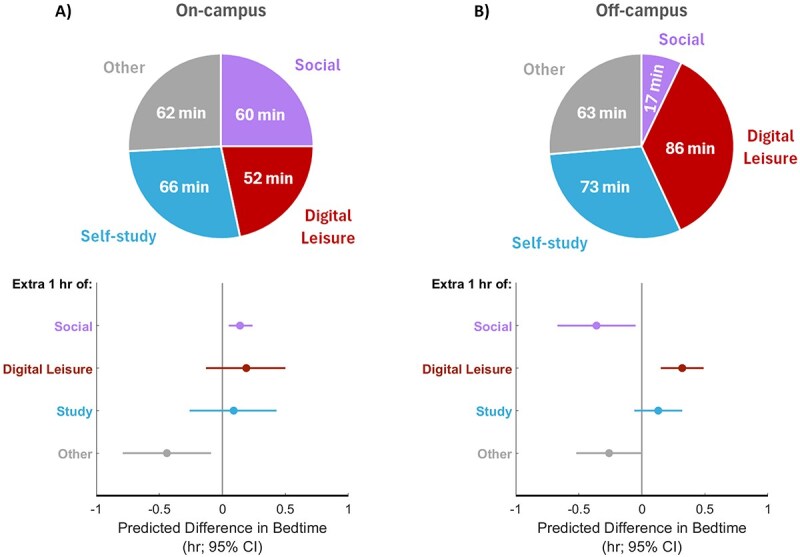
Late activities affect weekday bedtime differentially for on-campus and off-campus students. (A) Compositional data analyses showed that an extra hour of social activities (with proportional reduction in duration of other activities) would delay the bedtime of on-campus residents. (B) For the off-campus group, the same effect was seen with an increase in digital leisure. Reallocating 1 h to social activity was associated with earlier bedtime, presumably due to the concomitant reduction in screen time.

Time-use diaries showed that on-campus residents engaged in social and cocurricular activities late into the night, whereas off-campus students engaged in substantial digital leisure activities (Figure 5). During the 4 h preceding individual weekday bedtimes, the on-campus group (Figure 6A) spent similar amounts of time on social (60 min), Digital Leisure (52 min), self-study (66 min), and other (62 min), whereas the off-campus group (Figure 6B) spent the most time on digital leisure (86 min), compared to self-study (73 min) and other (63 min), and the least time on social (17 min). CoDA showed that bedtime would be delayed for on-campus students if social activities were increased by 1 h (with proportional reductions in other activity types). For the off-campus group, the same effect would be seen with an increase in digital leisure. Reallocating 1 h to social activity was associated with earlier bedtime, presumably due to the concomitant reduction in screen time. These results indicated that on-campus students with more social activities at night tended to sleep later, whereas off-campus students who spent more time on their digital devices slept later.

## Discussion

Sleep patterns in freshmen evolved during the semester: following an expected diminution in the first half of the semester [20,34], weekday sleep duration increased in the second half. We also found that situations expected to result in more sleep, e.g. not having to commute by staying on campus, were not associated with longer sleep. Unexpectedly, students appeared to skip classes—particularly those early in the morning—in order to catch up on sleep.

The uptrend in sleep duration in the latter part of the semester is surprising as freshmen might be expected to increasingly prioritize academic and social commitments, reducing sleep on weekdays and catching up on weekends [35,36]. Accordingly, the intention to improve health has motivated recent studies that incentivized college students to sleep longer [37,38]. However, contrary to expectation, our students showed a gradual extension of sleep duration on weekdays in the second half of the semester. This appears to be due to delayed wake times, afforded by students skipping early morning classes. Obtaining consistently adequate or nearly adequate sleep across weekdays and weekends supports optimal health outcomes for university students facing diverse demands [39].

Our findings are also counter-intuitive because sleep improvement interventions typically focus on sleeping earlier [40]. In our study, students did not change their bedtimes over the course of the semester. Instead, they extended their sleep by waking up later. Indeed, this pattern may reflect the propensity toward more eveningness in this age group in general [41–43], and also bears resemblance to the shifts observed during COVID-19 pandemic lockdowns, where the relative flexibility in schedules resulted in longer sleep, obtained by delaying wake times [44,45]. Similarly, these shifts in wake time were often observed despite participants reporting an aspiration to sleep earlier [46].

While the shift to later wake times resulted in longer sleep, other factors expected to relate to longer sleep did not show this association. Despite not having to commute and spending less late-night leisure time on digital devices (Figure 5), students living on campus still averaged about 20 min less weekday sleep than their off-campus counterparts. This resulted from later bedtimes and not utilizing enhanced opportunities to take more frequent or longer daytime naps. Later bedtimes and rise times are common with on-campus living and are associated with participation in nocturnal social activities [26,29]. Digital time-use diary information revealed that cocurricular and social activities delayed their weekday bedtimes. Scheduled weekday cocurricular and social engagements are integral to the incentive structure for retaining hostel residency, and these activities likely contribute to occupying weekday evening and late-night hours. In contrast, digital leisure activities delayed the bedtimes of those staying off-campus.

### Strengths and limitations

Broad and sustained adoption of the digital phenotyping tools used in this study (>70 per cent of participants providing Oura data over 20 weeks) underscores their acceptance by today’s digitally savvy students. The lack of seasonal effects [42] eliminates a common confound in extended longitudinal studies that focus on sleep duration and timing [23,47–49].

The unobtrusive, large-scale longitudinal study of sleep patterns and relevant factors influencing them provided insights that challenge common assumptions about how college students sleep. Increased sleep duration is favorable for health and well-being, but as illustrated here, may be achieved in unexpected ways. This can guide fresh practical interventions. For example, the scheduling of nighttime cocurricular activities to satisfy eligibility demands for on-campus residency could be avoided to allow students to get more sleep on-campus. In addition, early morning classes should be shifted later.

Although we did not prompt any behavioral change, we cannot exclude the possibility that participation heightened valuation of sleep through creating awareness of unhealthy sleep habits as students viewed their sleep data and reflected on their use of time in the course of providing data. In addition, lower completion rates were observed on the more time-consuming, self-report channels (e.g. EMA, time-use diaries) that were delivered later in the semester (Supplementary Methods). In theory, the reduced numbers contributing to these data could bias the results. However, the overall trends in sleep timing were similarly observed in both high and low compliance participants (Supplementary Figure S1), i.e. not likely to have been affected by missing data. The long-term health and well-being benefits of stabilizing weekday sleep duration remain to be determined. Finally, the study would have been enriched by collection of light and meal-timing data as these influence sleep.

## Conclusion

University freshmen who have relative autonomy to determine sleep timing, slept more by waking up later, often skipping early morning classes. Seemingly available opportunities to lengthen sleep were not taken. Our work highlights the utility of integrating smartphone-derived contextual information with sleep tracking data to uncover which of several routes to enabling adequate sleep are actually adopted.

## Supplementary Material

NUS1000_Wake_Time_SLEEP_Supplementary_2025Jun03_zsaf156

## Data Availability

The data in this article are available upon reasonable request from the authors.
