# Supplementary material for: A longitudinal study of sleep in university freshmen: facilitating and impeding factors
Source: Sleep. 2025 Jun 9;48(10):zsaf156. doi: 10.1093/sleep/zsaf156 (PMC12515602; doi:10.1093/sleep/zsaf156)
Supplement: NUS1000_Wake_Time_SLEEP_Supplementary_2025Jun03_zsaf156 [file nus1000_wake_time_sleep_supplementary_2025jun03_zsaf156.docx]

**A Longitudinal Study of Sleep in University Freshmen: Facilitating and Impeding Factors**

Chun Siong Soon^1^; Xin Yu Chua^1^; Ruth L. F. Leong^1^; Ju Lynn Ong^1^; Stijn A. A. Massar^1^; Shuo Qin^1^; Kyra H. M. Chong^1^; Jukka-Pekka Onnela^2^; Michael W. L. Chee^1^

1. Centre for Sleep and Cognition, Yong Loo Lin School of Medicine, National University of Singapore (NUS), Singapore

2. Department of Biostatistics, Harvard University, Boston, Massachusetts, USA

Corresponding author:

Dr. Michael W. L. Chee

Centre for Sleep and Cognition

NUS Yong Loo Lin School of Medicine,

MD1, 12 Science Drive 2

Singapore 117549

E-mail: [michael.chee@nus.edu.sg](mailto:michael.chee@nus.edu.sg)

**Supplementary Methods**

## Oura sleep timing filters

Up to one main sleep (sleep period with longest TST) was identified per day between 18:00 the previous day to 17:59 on the day in question. To accurately characterize the diversity of sleep patterns under free-living conditions, especially irregular or short sleep (including days without sleep), potential issues related to non-wear or other technical reasons needed to be considered. A day’s recorded main sleep was included/excluded based on: i) wear duration; ii) sleep timings; and iii) periods of wakefulness.

**Table S1.** **Wear duration filtering scheme for main sleep**

| **Longest Sleep Period (18:00 – 17:59)** | **Oura Ring Wear Duration** | |
| --- | --- | --- |
|  | **≥20h** | **<20h** |
| **TIB = 15h** | Excluded  (arbitrary upper limit; likely inaccurate) | |
| **3h ≤ TST < 15h** | Main Sleep | |
| **½h ≤ TST < 3h** | Main Sleep | Excluded (main sleep may have occurred during non-wear) |
| **No sleep detected** | Main Sleep = 0h |  |

When a day’s longest recorded TST was shorter than 3h, or no sleep was recorded, to ensure that the actual main sleep was not missed due to non-wear or other technical reasons, the wear duration of Oura Ring was considered, as specified in Table S1.

The attribution of a sleep period to a specific day may not be definitive as unconventional sleep hours can occur under naturalistic conditions. To mitigate such uncertainty in day attribution, especially for sleep episodes centered around 18:00, we further excluded from main sleep analyses those days with main sleep starting before noon the previous day or ending after 1800h on the day in question.

Sleep detection algorithms need to accommodate bouts of wakefulness within a sleep episode, i.e., WASO. However, if the maximum allowed WASO duration is too long, multiple sleep episodes in close proximity may be inappropriately concatenated, leading to an overestimation of TIB. We additionally considered an individual’s typical sleep timings when identifying such main sleep periods for exclusion from main sleep analyses: i) any 2h window containing a high proportion of wakeful movement (>1.5h), and ii) uncharacteristic sleep timings (bedtime earlier than median –1.5 IQR bedtime, or waketime later than median +1.5 IQR waketime).

## Compositional Data Analyses (CoDA)

CoDA was used to model four activity types, ‘Social’, ‘Digital Leisure’, ‘Self-study’ and ‘Others’, in the 4-hour period preceding individual bedtimes (R package ‘epicoda’^1^). Compositional exposures were expressed using isometric log-ratio (ilr) coordinates, a log-ratio transformation that allows standard statistical methods to be applied to compositional data. The ilr coordinates were constructed using pivot coordinates, commonly used in behavioral research due to their interpretability.

For a composition x = (x₁, x₂, x₃, x₄), the ilr transformation yields three coordinates:

- **ilr₁** = $\sqrt{\frac{3}{4}}$ $ln(\frac{\mathbf{x₁}}{\sqrt[3]{\mathbf{x₂}\times\boldsymbol{x₃\times x₄}}})$
- **ilr₂** = $\sqrt{\frac{2}{3}}$ $ln(\frac{\mathbf{x₂}}{\sqrt[2]{\boldsymbol{x₃\times x₄}}})$
- **ilr₃** = $\sqrt{\frac{1}{2}}$ $ln(\frac{\mathbf{x₃}}{\mathbf{x₄}})$

These log-ratios captured relative reallocations of time between activities while accounting for the constrained nature of time-use data^1,2^. Model outputs were visualized separately for the On-campus and Off-campus groups using plots showing changes in bedtime associated with hypothetical reallocations of time (e.g., adding 1 hour to social activities while proportionally reducing time from all others). All effects were interpreted relative to the mean composition of each sample, representing the expected bedtime change for an average individual when reallocating time between activities.

## On-campus accommodation allocation

The National University of Singapore, where this study was conducted, provides a variety of student accommodation within its campus. Freshmen are encouraged to apply for on-campus accommodation, though a place is not guaranteed. Financial aid schemes, including scholarships, bursaries and loans, are available for those who need help with hostel fees.

Singapore is a small city state (50km from east to west, 27km from north to south) with an efficient public transportation system. Thus, it is not uncommon for students to stay in their family homes, and commute to school.

**Questionnaire Scheduling**

Questionnaires were delivered in 3 survey sets across the 20-week study period. The following list of questionnaires were administered:

**Survey Set 1 (Week 1-2)**

- Pittsburgh Sleep Quality Index (PSQI)^3^
- Beck’s Anxiety Inventory (BAI)^4^
- Beck’s Depression Inventory (BDI)^5^
- Ford Insomnia Response to Stress Test (FIRST)^6^
- Bedtime Sleep Delay Questionnaire (in-house questionnaire)
- Barriers to Sleep Questionnaire (in-house questionnaire)

**Survey Set 2 (Week 7-8)**

- Brief-COPE^7^
- Big5 Personality Inventory^8^
- Insomnia Severity Index (ISI)^9^
- Oldenberg Burnout Inventory-Student Version^10^
- Pittsburgh Sleep Quality Index (PSQI)
- Perceived Stress Reactivity Scale^11^
- Barriers to Sleep Questionnaire (in-house questionnaire)
- Bedtime Sleep Delay Questionnaire (in-house questionnaire)
- Sleep Adequacy Questionnaire (in-house questionnaire)

**Survey Set 3 (Week 15-16)**

- Morningness-Eveningness Questionnaire (MEQ)^12^
- Insomnia Severity Index (ISI)
- Oldenberg Burnout Inventory-Student Version
- UCLA Loneliness Scale^13^
- Barriers to Sleep Questionnaire (in-house questionnaire)
- Bedtime Sleep Delay Questionnaire (in-house questionnaire)

# Supplementary Results

**Table S2. Mean (standard deviation) of weekend and weekday sleep timing and duration across academic calendar**

|  |  | **Instructional (Weeks 1-6)** | **Instructional**  **(Weeks 9-14)** | **Reading** | **Examination** | **Vacation** |
| --- | --- | --- | --- | --- | --- | --- |
| **Waketime** | **Weekend** | 09:37  (85min) | 09:41  (97min) | 09:47  (109min) | 09:43  (108min) | 09:57  (117min) |
|  | **Weekday** | 08:44  (65min) | 08:56  (76min) | 09:24  (93min) | 09:10  (80min) | 09:43  (99min) |
|  | **Difference** | 53min  (55min) | 45min  (71min) | 23min  (91min) | 34min  (88min) | 14min  (93min) |
| **Bedtime** | **Weekend** | 01:58  (81min) | 02:03  (87min) | 02:02  (106min) | 02:07  (99min) | 02:02  (108min) |
|  | **Weekday** | 01:41  (73min) | 01:52  (79min) | 01:59  (90min) | 01:53  (83min) | 01:51  (95min) |
|  | **Difference** | 17min  (46min) | 12min  (56min) | 4min  (78min) | 14min  (72min) | 12min  (76min) |
| **Total Sleep Time** | **Weekend** | 6h 49min  (49min) | 6h 48min  (57min) | 6h 54min  (62min) | 6h 46min  (59min) | 7h 00min  (68min) |
|  | **Weekday** | 6h 18min  (40min) | 6h 19min  (43min) | 6h 36min  (51min) | 6h 29min  (45min) | 6h 59min  (49min) |
|  | **Difference** | 30min  (48min) | 29min  (54min) | 18min  (62min) | 18min  (59min) | 1min  (67min) |

## Similar waketime trends for freshmen with different participation rates


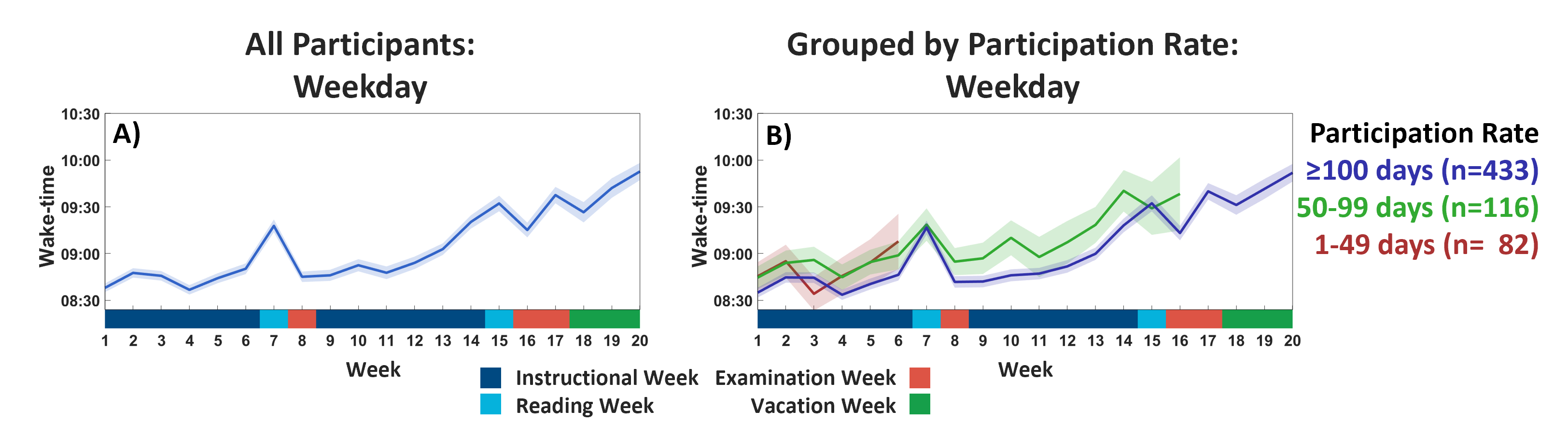


**Fig. S1. Waketimes of freshmen with different completion rates across semester.** Similar trends of increasingly delayed weekday waketimes as the semester progressed were observed in participants with different participation rates. (Any week with <30 participants in a group was excluded.)

## Sleep quality across the semester


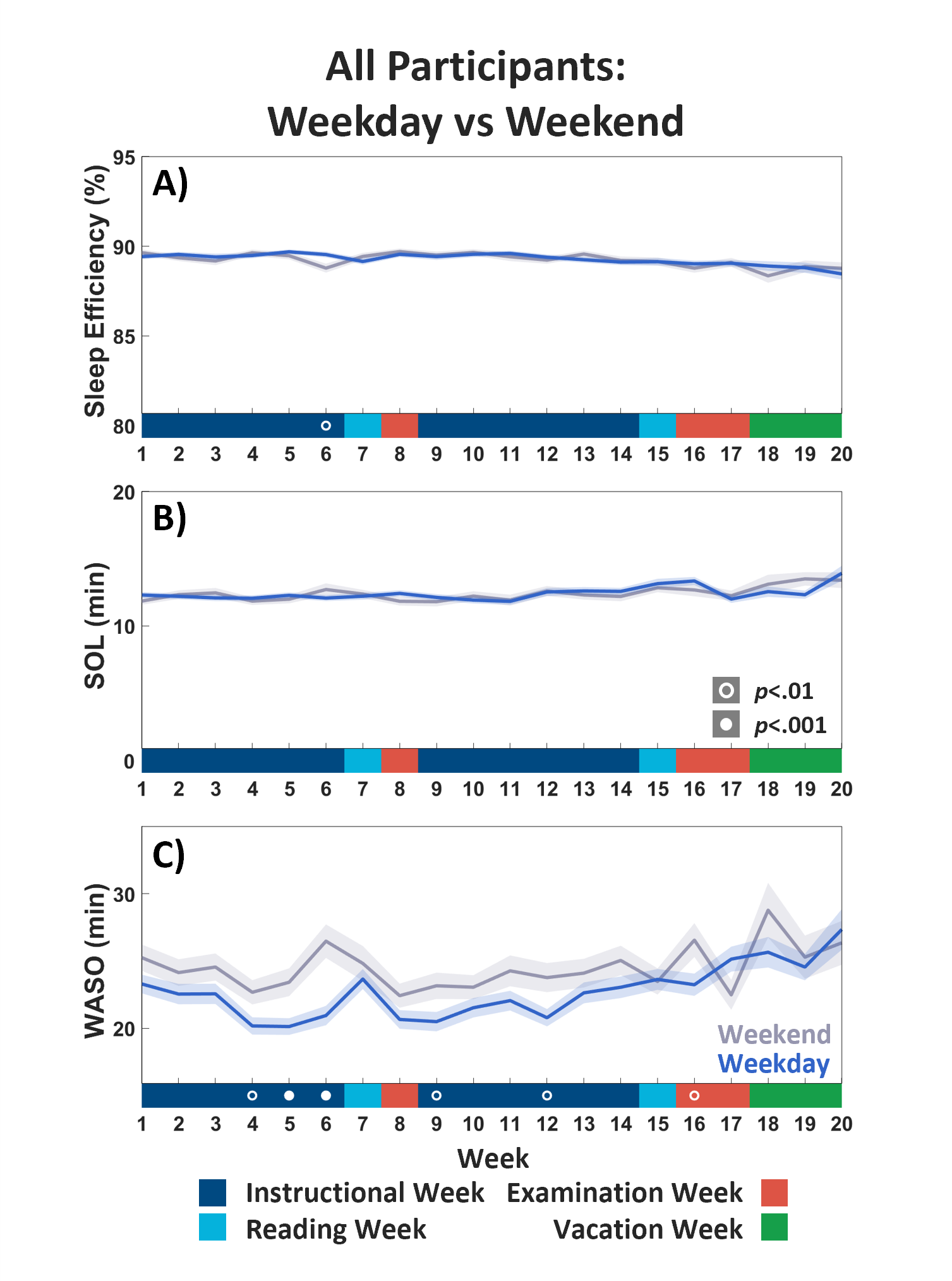


**Fig. S2. Additional sleep dimensions across first semester: (A) sleep efficiency; (B) sleep onset latency (SOL) and wake-after-sleep-onset (WASO).** Sleep efficiency and SOL remained stable, whereas changes in WASO were similar to fluctuations in sleep duration (Fig. 3C). Error bands represent standard error of the mean (SEM). Open (*p*<.01) and closed (*p*<.001) circles indicate significant differences (t-tests, uncorrected) for each week.

## Defining first class of the day based on time-use diaries


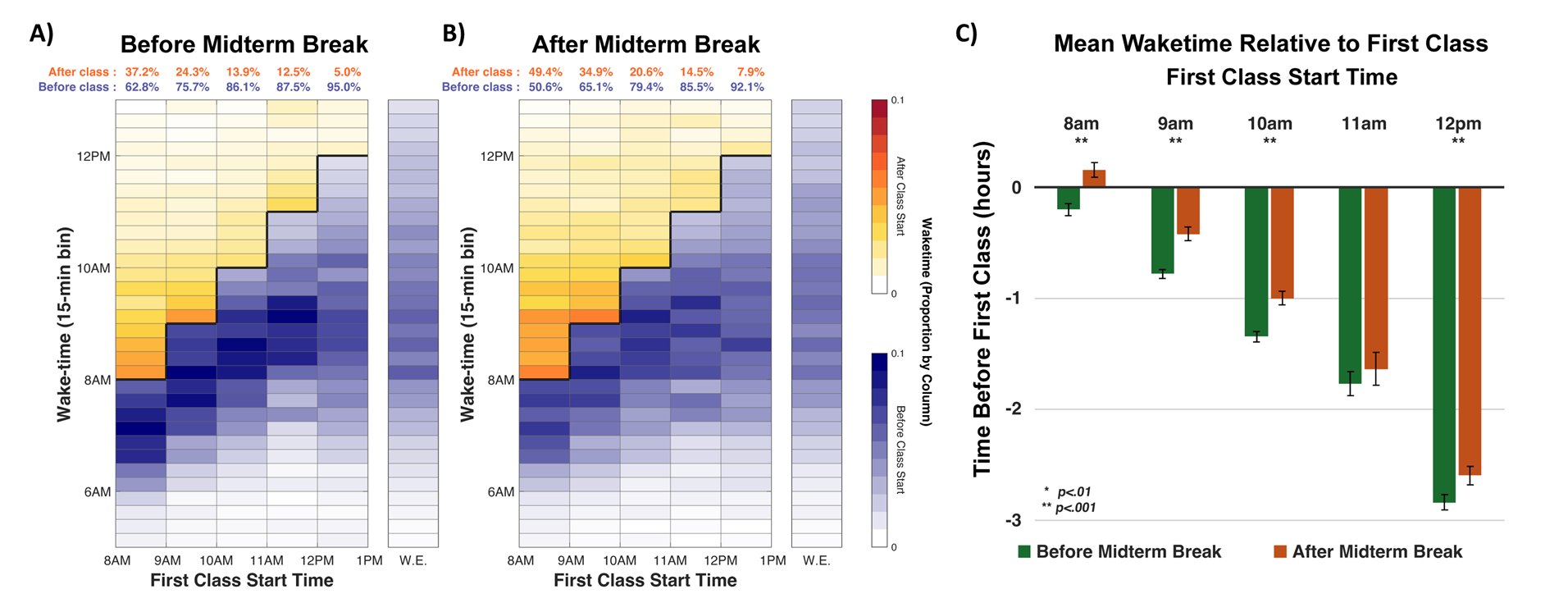


**Fig. S3. Waketime in relation to first class of the day as inferred from time-use diaries.** As individual class schedule was largely similar across Instructional weeks, the first class start time for each day of the week was based on the earliest reported “Lessons” on Instructional weekdays. Similar trends were observed as in Fig. 4, whereby first class timings were based on official timetables.

## Differences in sleep and activity patterns between On- and Off-campus freshmen


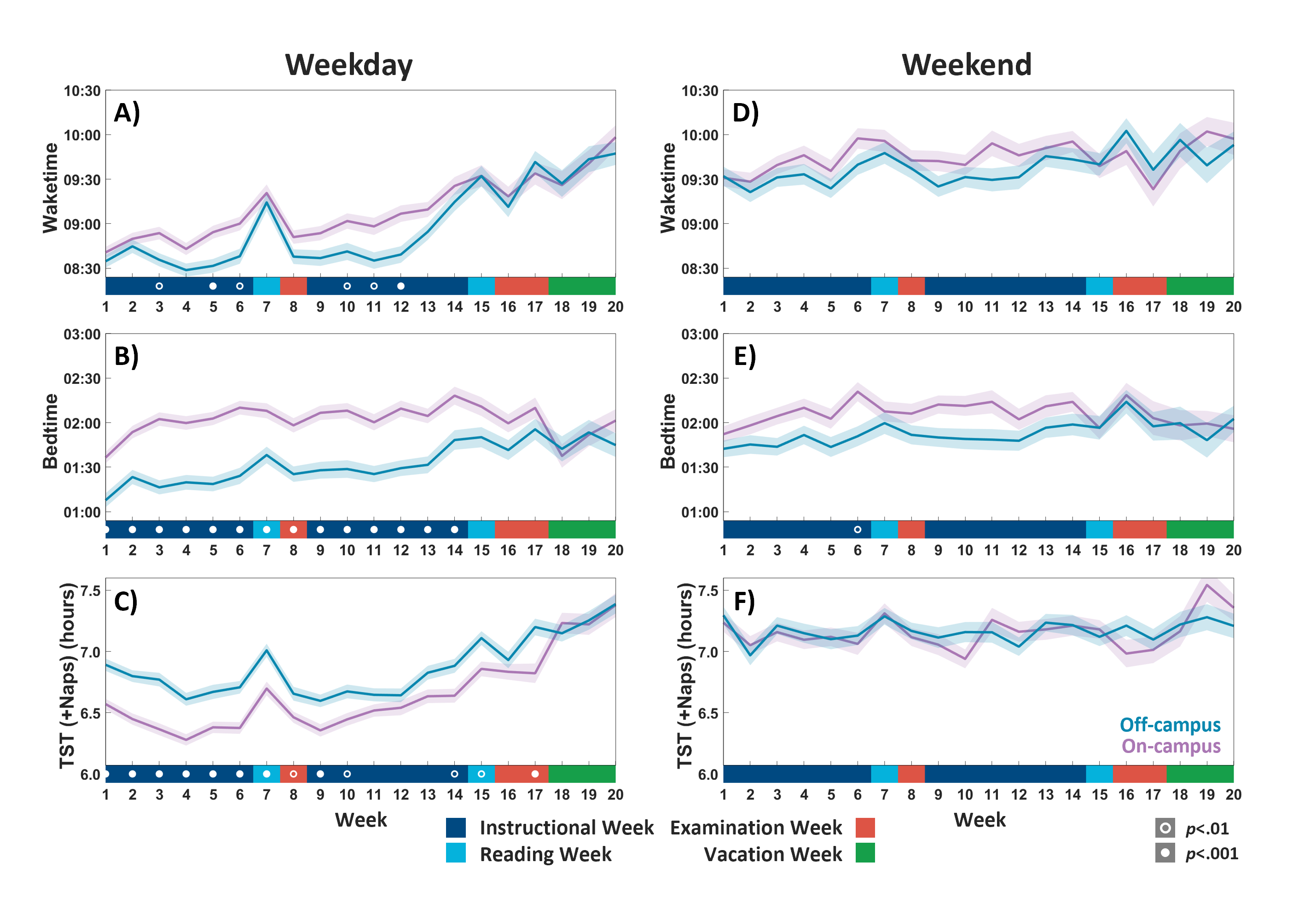


**Fig. S4. Association between residence and sleep patterns.** (A–C) On weekdays, residence was associated with differences in sleep timing and duration, even when naps were included. (D–F) On weekends, differences in sleep timing were reduced. No difference was found in sleep duration. Both groups showed stable weekend sleep timing and duration across the semester.

**References**

1. Walmsley R, Chan S, Smith-Byrne K, et al. Reallocation of time between device-measured movement behaviours and risk of incident cardiovascular disease. Br J Sports Med. 2021; 56 (18): 1008-1017.

2. Dumuid D, Pedisic Z, Palarea-Albaladejo J, Martin-Fernandez JA, Hron K, Olds T. Compositional Data Analysis in Time-Use Epidemiology: What, Why, How. Int J Environ Res Public Health. 2020; 17 (7).

3. Buysse DJ, Reynolds CF, 3rd, Monk TH, Berman SR, Kupfer DJ. The Pittsburgh Sleep Quality Index: a new instrument for psychiatric practice and research. Psychiatry Res. 1989; 28 (2): 193-213.

4. Beck AT, Epstein N, Brown G, Steer R. *Beck Anxiety Inventory.* APA PsycTests; 1988.

5. Beck AT, Ward CH, Mendelson M, Mock J, Erbauch J. *Beck Depression Inventory (BDI)* APA PsycTests; 1961.

6. Kalmbach DA, Pillai V, Arnedt JT, Drake CL. Identifying At-Risk Individuals for Insomnia Using the Ford Insomnia Response to Stress Test. Sleep. 2016; 39 (2): 449-456.

7. Carver CS. You want to measure coping but you protocol’s too long: Consider the brief COPE. International Journal of Behahvioral Medicine. 1997; 4: 92-100.

8. John OP, Donahue EM, Kentle RL. *Big Five Inventory.* APA PsycTests; 1991.

9. Morin CM, Belleville G, Belanger L, Ivers H. The Insomnia Severity Index: psychometric indicators to detect insomnia cases and evaluate treatment response. Sleep. 2011; 34 (5): 601-608.

10. Reis D, Xanthopoulou D, Tsaousis I. Measuring job and academic burnout with the Oldenburg Burnout Inventory (OLBI): Factorial invariance across samples and countries. Burnout Research. 2015; 2 (1): 8-18.

11. Schlotz W, Yim IS, Zoccola PM, Jansen L, Schulz P. The Perceived Stress Reactivity Scale: measurement invariance, stability, and validity in three countries. Psychol Assess. 2011; 23 (1): 80-94.

12. Horne JA, Ostberg O. A self-assessment questionnaire to determine morningness-eveningness in human circadian rhythms. Int J Chronobiol. 1976; 4 (2): 97-110.

13. Hughes ME, Waite LJ, Hawkley LC, Cacioppo JT. A Short Scale for Measuring Loneliness in Large Surveys: Results From Two Population-Based Studies. Res Aging. 2004; 26 (6): 655-672.
